# Supplementary material for: An Integrated Proteomic and Glycoproteomic Investigation Reveals Alterations in the N-Glycoproteomic Network Induced by 2-Deoxy-D-Glucose in Colorectal Cancer Cells
Source: Int J Mol Sci. 2022 Jul 26;23(15):8251. doi: 10.3390/ijms23158251 (PMC9331968; doi:10.3390/ijms23158251)

Integrated Proteomic and Glycoproteomic Investigation Reveals Alterations in *N*-Glycoproteomic Network Induced by 2-Deoxy-D-Glucose in Colorectal Cancer Cells

Cheng Ma[1*], Hong-Yuan Tsai[2], Qi Zhang[3], Lakmini Senavirathna[1], Lian Li[3], Lih-Shen Chin[3], Ru Chen[2], Sheng Pan[1,4*]

[1] The Brown Foundation Institute of Molecular Medicine, [4] Department of Integrative Biology and Pharmacology, University of Texas Health Science Center at Houston, Houston, TX 77030, USA

[2] Division of Gastroenterology and Hepatology, Department of Medicine, Baylor College of Medicine, Houston, TX 77030, USA

[3] Department of Pharmacology and Chemical Biology, Emory University School of Medicine, Atlanta, GA 30322, USA

*Correspondence: sheng.pan@uth.tmc.edu

cheng.ma@uth.tmc.edu

Figure S1. GO-enrichment of cellular functions based on down-regulated proteins in HT29 cell induced by 2DG.

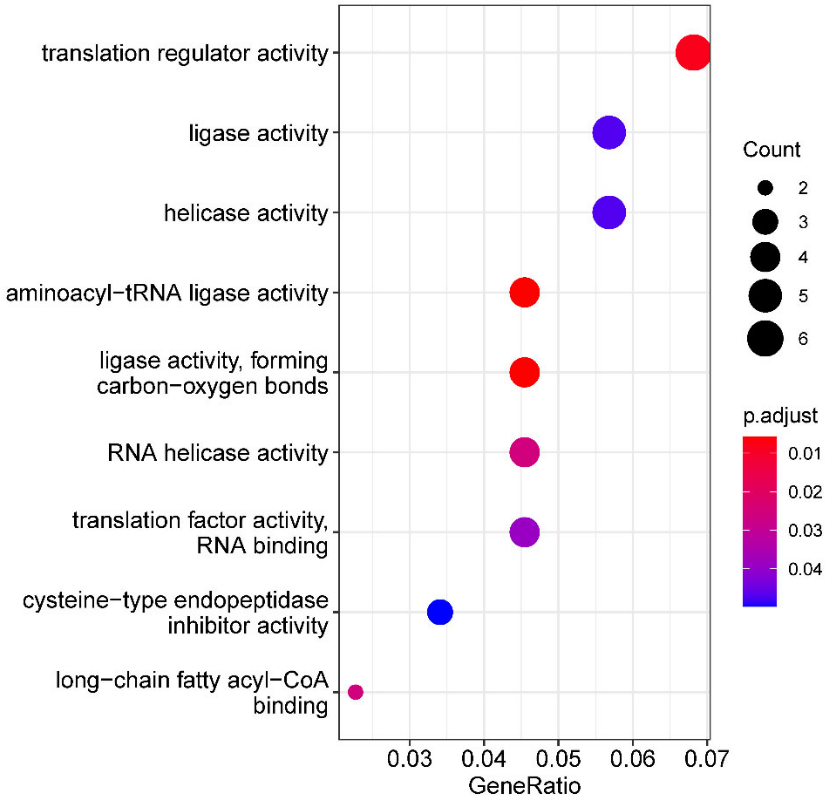

Figure S2. Comparison of glycoforms with and without 2DG treatment based on the saccharide types, including glycans containing oligomannose, fucose, and sialic acid. The result indicates that the abundance of oligomannose-type *N*-glycan has the most decreases compared to sialic acid and fucose-containing N-glycans. *Ratio**: normalization with the N-glycopeptides of internal standard bovine THYG.

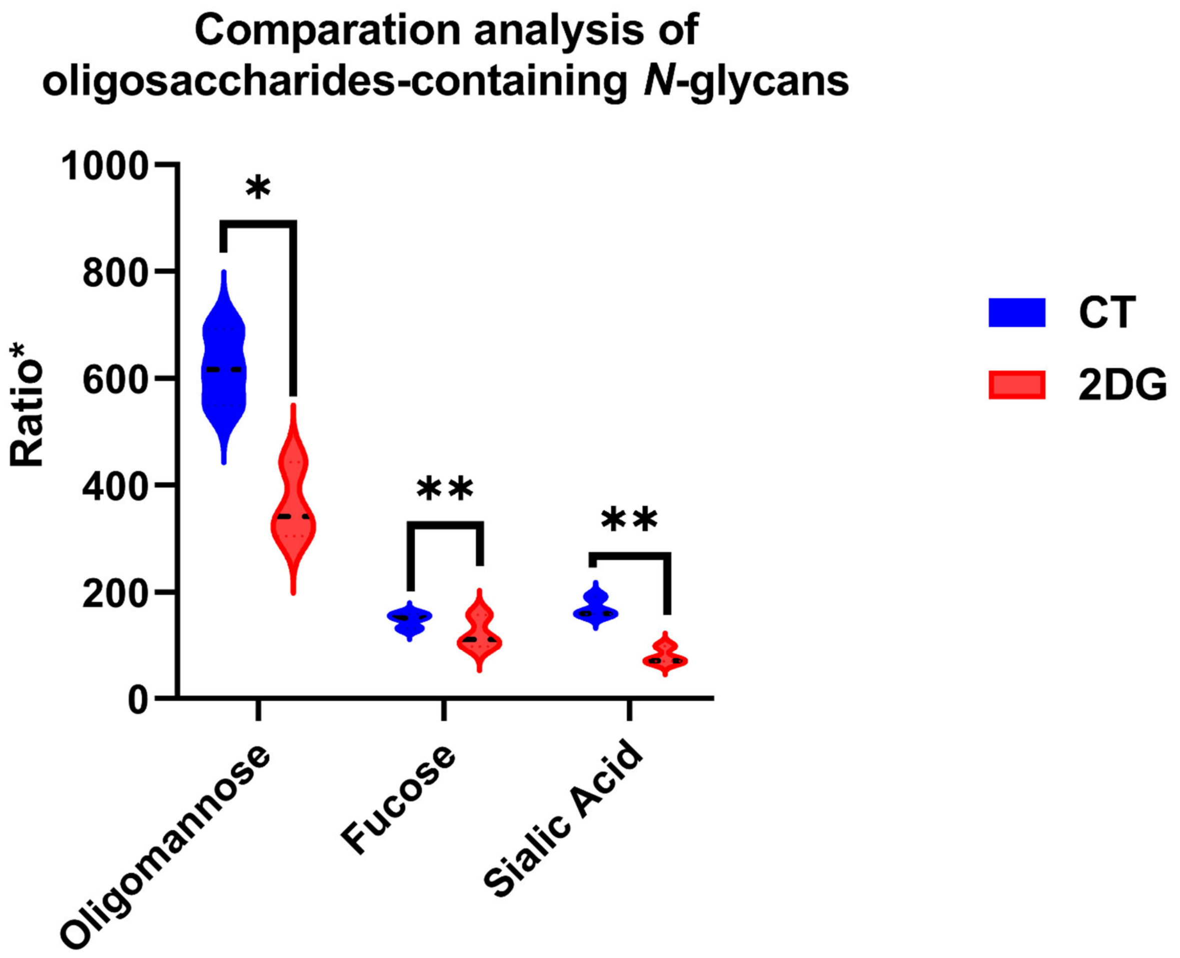

Figure S3. Comparison of protein expressional profile with the changes in N-glycosylation induced by 2DG. Venn analysis of differentially expressed proteins with dysregulated N-glycoproteins: up-regulation (top) and down-regulation (down).

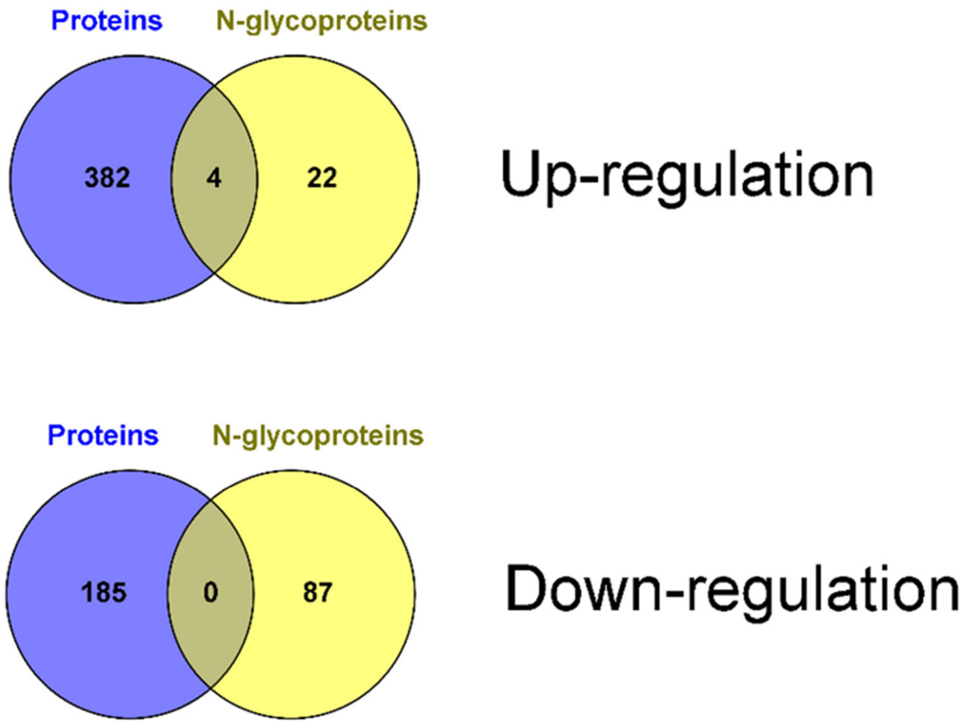

Supplement: Supplementary file 1 [file ijms-23-08251-s001.zip › Supplemental.pdf]
